# PEG-IFN Alpha but Not Ribavirin Alters NK Cell Phenotype and Function in Patients with Chronic Hepatitis C

**DOI:** 10.1371/journal.pone.0094512

**Published:** 2014-04-21

**Authors:** Antoaneta A. Markova, Ulrike Mihm, Verena Schlaphoff, Sebastian Lunemann, Natalie Filmann, Birgit Bremer, Thomas Berg, Christoph Sarrazin, Stefan Zeuzem, Michael P. Manns, Markus Cornberg, Eva Herrmann, Heiner Wedemeyer

**Affiliations:** 1 Department of Gastroenterology, Hepatology and Endocrinology, Hannover Medical School, Hannover, Germany; 2 Department of Medicine 1, JW Goethe-University Hospital, Frankfurt am Main, Germany; 3 Institute of Biostatistics and Mathematical Modelling, Faculty of Medicine, JW Goethe-University, Frankfurt am Main, Germany; 4 Department of Internal Medicine, Division of Gastroenterology and Rheumatology, University of Leipzig, Leipzig, Germany; University of Washington, United States of America

## Abstract

**Background:**

Ribavirin (RBV) remains part of several interferon-free treatment strategies even though its mechanisms of action are still not fully understood. One hypothesis is that RBV increases responsiveness to type I interferons. Pegylated Interferon alpha (PEG-IFNa) has recently been shown to alter natural killer (NK) cell function possibly contributing to control of hepatitis C virus (HCV) infection. However, the effects of ribavirin alone or in combination with IFNa on NK cells are unknown.

**Methods:**

Extensive ex vivo phenotyping and functional analysis of NK cells from hepatitis C patients was performed during antiviral therapy. Patients were treated for 6 weeks with RBV monotherapy (n = 11), placebo (n = 13) or PEG-IFNa-2a alone (n = 6) followed by PEG-IFNa/RBV combination therapy. The effects of RBV and PEG-IFNa-2a on NK cells were also studied in vitro after co-culture with K562 or Huh7.5 cells.

**Results:**

Ribavirin monotherapy had no obvious effects on NK cell phenotype or function, neither ex vivo in patients nor in vitro. In contrast, PEG-IFNa-2a therapy was associated with an increase of CD56^bright^ cells and distinct changes in expression profiles leading to an activated NK cell phenotype, increased functionality and decline of terminally differentiated NK cells. Ribavirin combination therapy reduced some of the IFN effects. An activated NK cell phenotype during therapy was inversely correlated with HCV viral load.

**Conclusions:**

PEG-IFNa activates NK cells possibly contributing to virological responses independently of RBV. The role of NK cells during future IFN-free combination therapies including RBV remains to be determined.

## Introduction

Persistent hepatitis C virus (HCV) infection affects about 160–180 million individuals worldwide [1]. Hepatitis C is one of the main causes of end stage liver disease and hepatocellular carcinoma (HCC). The disease burden caused by HCV is expected to increase during the next years despite significant progress in antiviral therapy options [2].

The immunopathogenesis of chronic hepatitis C is still not completely understood - almost 25 years after the discovery of HCV. An important role of T cell responses to control early acute HCV infection is well established. Various mechanisms how the virus evades the adaptive immune system have been suggested, including viral evolution leading to T cell escape, functional exhaustion of T cells, increased frequencies of regulatory T cells, impaired CD4 T cell help and direct interference of HCV with antigen presenting cells [3], [4]. Beyond T cell responses, the role of natural killer cells (NK cells) in hepatitis C virus infection has received increasing attention in recent years. NK cells are able to control viral infections by either inhibiting replication through cytokine synthesis or through direct elimination of infected cells. The activity of NK cells is regulated by a fine tuned balance between activatory and inhibitory receptors on their cell surface. Distinct combinations of specific killing inhibitory receptors (KIR), specific HLA class I molecules and their respective ligands were associated with either spontaneous clearance or chronicity of acute HCV infection [5], [6]. Moreover, NK cell phenotype and function have been shown to be altered both in acute and chronic hepatitis C [7], [8] and linked with either outcome of acute infection [9]–[11] or response to antiviral therapy [12]–[14].

The current standard treatment of chronic hepatitis C still includes administration of pegylated interferon alfa (PEG-IFNa) and ribavirin (RBV) [15]. In addition, HCV protease inhibitors (PIs) have been approved for HCV genotype 1 infection by FDA and EMA in 2011 [16]. HCV PIs are currently used in combination with PEG-IFNa and RBV and between 60 to 90% of patients can be successfully treated with this triple therapy. However, many patients still do not clear HCV. Response rates are in particular poor in patients with advanced liver disease [17]. Additionally, a significant proportion of HCV-infected individuals cannot be treated at all with PEG-IFNa and/or new PIs due to contraindications and co-morbidities [18]. IFN free combination therapies with different direct acting antivirals will likely become available during the next few years. Of note, RBV is still part of many new treatment regimes in clinical development (www.clinicaltrials.gov).

The detailed mode of action how IFNa and RBV provoke their antiviral effects against HCV are not completely understood. IFNa induces distinct expression of interferon stimulated genes (ISGs) which in the end leads to suppression of viral replication [19]. In addition, IFNa is believed to cause immunomodulatory effects by acting on various cells of the innate and adaptive immune system. We recently showed that IFNa induces expression of tumor necrosis factor related apoptosis inducing ligand (TRAIL) on NK cells which may contribute to control of viral replication [20]. TRAIL expression on NK cells correlated with the phase 2 decline during antiviral combination therapy [20]. However, the effects of RBV in the context of antiviral therapy of HCV infection are rather unclear. Among various suggested possible modes of action [21] ribavirin is believed to alter immune responses. For example shifts in the TH1/2 cytokine profiles have been reported both in animal models [22] as well as during ribavirin treatment of hepatitis C in humans [23]. Ribavirin is believed to increase the susceptibility of hepatocytes to IFNa stimulation [24]. Nevertheless, it is unknown if RBV may also have distinct effects on NK cells, either alone or in combination with IFNa.

We had the unique chance to study peripheral blood mononuclear cell (PBMC) samples obtained from patients during a clinical trial investigating the effects of a six-week lead-in therapy with either RBV alone, PEG-IFNa alone or placebo on the early HCV RNA kinetics [25]. We therefore aimed to address the question if RBV alters the phenotype and function of human NK cells in vivo, alone or in combination with PEG-IFNa.

## Materials and Methods

### Patients and Treatment

Samples were obtained as part of a prospective, randomised, partially placebo controlled clinical trial which has been described in detail previously [25]. In brief, patients with chronic HCV genotype 1 infection were treated for six weeks with either RBV monotherapy (group A, RBV 1000–1200 mg/d), placebo (group B) or PEG-IFNa alone (group C, Peginterferon alpha-2a 180 µg/week), followed by twelve weeks of PEG-IFNa-RBV combination. Subsequent antiviral treatment after the 12 weeks of therapy within this trial was administered for a total treatment duration of up to 48 weeks according to the German guidelines and under the responsibility of the investigators [25]. The initial aim of the study was to investigate early kinetics of HCV RNA in the different study arms. Blood samples were taken frequently during the first days of therapy, peripheral blood mononuclear cells (PBMC) were isolated before therapy and after 2, 14, 28, 42, 44, 56, 70, 84 and 126 days of therapy. Standard of care (SOC) was continued after week 12 according to the national treatment guidelines [26]. Patients gave written informed consent for the investigation of immunological parameters as part of the study protocol that has been approved by the ethics committee of the participating university hospitals (principal ethics committee of the medical association of Saarland and the ethics committees of the university hospitals in Berlin, Frankfurt and Hannover).

Blood samples were drawn in CDTA (citrate dextrose) tubes, peripheral blood mononuclear cells (PBMC) were separated using standard Ficoll Density Centrifugation method, washed 3 times with phosphate-buffered saline (PBS) and cryopreserved. After thawing, cells were tested for viability with trypan blue and considered for further experiments if viability was more than 90%. Altogether, vital cells were available from 30 patients: 11 in group A, 13 in group B and 6 in group C. Summary of patient characteristics are shown in Table 1 and characteristics of each individual in Table S1.

**Table 1:** Patient epidemiological and clinical characteristics at baseline (day 0).

Group	Therapyd0–d42	Therapy d42–d126	Mean age, years	Mean HCV RNA, log10	Media ALT, IU/ml	Median AST, IU/ml	Gender, male/female	Mean BMI, kg/m^2^
**Group A** (n = 11)	RBV	PEG-IFN RBV	48±10	6.1±0.9	53 (31–179)	46 (26–241)	4/7	27±5.2
**Group B** (n = 13)	Placebo	PEG-IFN RBV	51±15	6.1±0.9	65 (32–206)	63 (30–163)	4/9	26±4.1
**Group C** (n = 6)	PEG-IFN	PEG-IFN RBV	50±15	6.9±0.4	96 (57–176)	65 (38–203)	4/2	25±3.3

*ALT - alanin aminotransferase, AST - aspartate aminotransferase, BMI - body mass index.

### Analysis of NK Cell Phenotype and Function in Patients

NK cell phenotype and function was determined as previous described [20], [27]. Detailed protocols and antibodies used in this study are provided in the Supporting Information.

### Effects of Ribavirin on NK Cells in vitro

To investigate the effects of ribavirin on NK cells in vitro, NK cells from healthy blood donors were studied. Freshly isolated PBMC were cultured at 2.5×10^6^ cells/ml at 37°C in complete RPMI medium alone and/or stimulated with K562 or Huh7.5 target cells. Additionally, cells were stimulated with RBV in a concentration of 0.5 µg/mL, 5 µg/ml and 50 µg/ml alone or in combination with PEG-IFN-alfa-2a in a concentration of 100 ng/mL or in combination with IL-12/IL-15.

### Statistical Analyses

To evaluate the dynamics of the different immunological markers, we applied a mixed effects model with piecewise log-linear regression function for the respective treatment phases. In this way it is possible to differentiate between PEG-IFN-alfa-2a and RBV effects and the corresponding tests are more powerful than paired sample tests ignoring intermediate measurements. Nevertheless, some results for paired t-tests are also given additionally. An additional mixed effect regression model including HCV RNA was used to evaluate the effect of viral load on the different markers. Spearman rank correlations were calculated to evaluate the associations between the immunological markers and viral kinetic parameters as interferon efficacy which were previously calculated [25]. In short, the underlying mathematical model uses a standard biphasic nonlinear ordinary differential equation system accounting for varying drug levels of PEG-IFN-alfa-2a in treatment phases including PEG-IFN-alfa-2a. Estimates for mean and maximal efficacy of PEG-IFN-alfa-2a in blocking viral production and efficacy of RBV in rendering part of the newly produced virus as non-infectious are provided as well as estimates of the infected cell loss rate. All statistical tests were two-sided with a significance level of a = 5% and account appropriately for dependencies between samples from the same patients. We used R software (R Foundation of Statistical Computing, Vienna, Austria) and the nlme package. Figures were generated using Graph Pad Prism 5 Software, Version 5.01.

## Results

### NK Cell Frequencies during Monotherapy with PEG-IFNa or Ribavirin and Combination Antiviral Therapy

**Figure 1:**
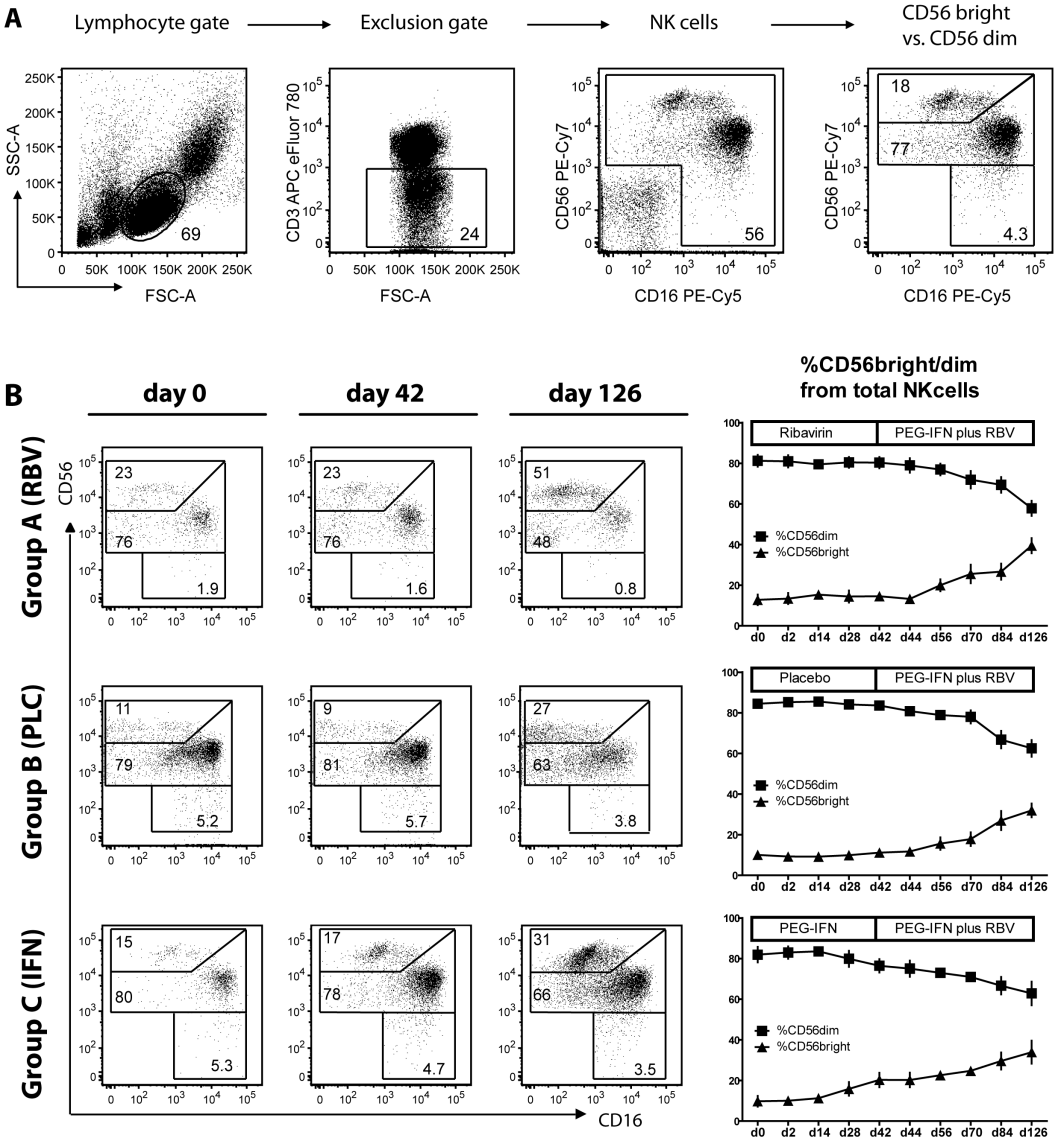
Frequency of NK cell subsets during therapy. (A) Gating strategy to distinguish NK cells and subgroups CD56^dim^ and CD56^bright^ cells. (B) Representative FACS plots for d0, d42 and d126 stainings and mean values for all patients are shown. d, day; RBV, Ribavirin; PLC, Placebo; PEG-IFN, pegylated Interferon.

In order to study the effects of RBV or PEG-IFNa monotherapy and the combination of both on NK cells, we first assessed frequency and phenotype of NK cells directly ex vivo in peripheral blood at several timepoints during antiviral therapy. NK cells were identified as CD3-negative, CD56 and/or CD16 positive cells after gating on lymphocytes (Fig. 1A). Gating strategies to distinguish NK cells with low and high expression levels of CD56 (CD56^dim^ vs. CD56^bright^ cells) are also shown in Figure 1A.

The frequency of NK cells was not affected by therapy in any of the three treatment groups (data not shown). However, administration of PEG-IFNa but not of ribavirin led to a shift in the frequency of NK cell subtypes (Fig. 1B). While the relative proportion of CD56^dim^ and CD56^bright^ cells did not change during ribavirin monotherapy (p>0.05), a marked increase in CD56^bright^ (p<0.0001) and a decline of CD56^dim^ (p<0.0001) cells was seen after the addition of PEG-IFNa until day 126 of therapy in group A patients (e.g., CD56^bright^ day 42∶15±2.4% vs. day 126∶39±3.7%, p<0.0001). A comparable change in the CD56^dim^/CD56^bright^ ratio occurred after day 42 in patients receiving first placebo (group B) (Fig. 1B, p<0.0001). PEG-IFNa monotherapy also induced an increase of the CD56^bright^ population (p<0.0001 in the regression model; e.g., 9.8%±2.6 at baseline vs. 20%±3.5 at day 42 but not significant for paired comparison because of missing paired samples) which further augmented during subsequent combination therapy (day 126∶34%±5.7, p<0.004 for a paired comparison with day 0).

Thus, PEG-IFNa therapy was associated with a 2–3 fold increase of CD56^bright^ cells within 12 weeks of therapy. Ribavirin had no influence on the relative CD56^dim^ vs. CD56^bright^ ratio and ribavirin pre-treatment did not alter the PEG-IFNa effect on NK cell subsets.

### PEG-IFNa but not Ribavirin Induces an Activated Phenotype of NK Cells

**Figure 2:**
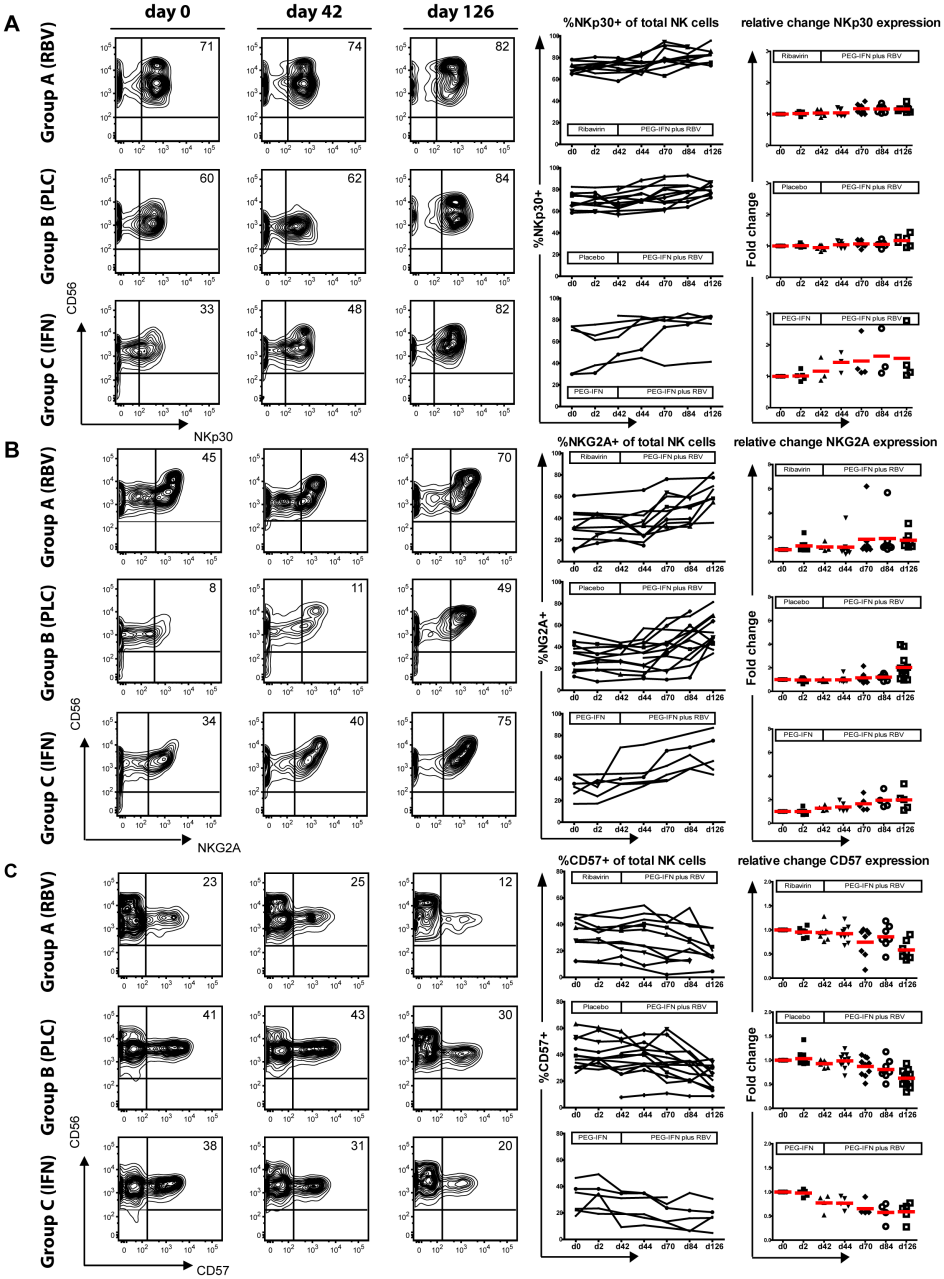
Phenotypical changes during treatment with RBV, PEG-IFNa and combination therapy. PBMC from HCV patients were stained directly ex vivo. Representative FACS plots for d0, d42 and d42 and single patient courses for (A) NKp30 (B) NKG2A and (C) CD57 expression on total NK cells are shown. Percentage of positive cells as well as fold change compared to baseline expression are represented.

We next aimed to investigate to what extent different treatment regimens may alter NK cell phenotypes as suggested by other studies [12], [14]. Of note, ribavirin monotherapy had no effect on expression levels of NKp30 on total NK cells with stable frequencies of NKp30-positive cells until day 42 in group A (Fig. 2A) which held true for both CD56^dim^ and CD56^bright^ NK cells (Fig. S1). After adding PEG-IFNa, the percentage of NKp30-positive cells increased in all patients with mean values of 72±2.1% at day 42 and 82±2.6% at day 126 (p = 0.006, paired sample test). Almost similar values of NKp30 expression were observed in patients during combination therapy in group B (placebo until day 42) with a mean increase from 68±2.9% to 80±1.9% (p = 0.005 for the increase in group A and B in the regression model and no significant differences between both groups). PEG-IFNa monotherapy also induced a significant increase in NKp30-positive cells until day 42 (p<0.001) with further enhancement of NKp30 expression during subsequent combination treatment (Fig. 2A and Table 2). Changes of NKp30 expression during therapies were comparable for both CD56^dim^ and CD56^bright^ cells (Fig. S1).

**Table 2:** Change of NK cell frequency, phenotype and function during monotherapy (PEG-IFNa or RBV) and combination therapy.

Parameter	PEG-IFNa alone (Group C d0–d42)	RBV alone (Group A d0–d42)	PEG-IFNa-RBV after placebo (Group B d42–d126)
% NK cells	↔	↔	↔
%CD56^bright^ cells	↑↑↑	↔	↑↑↑
%CD56^dim^ cells	↓↓↓	↔	↓↓↓
%NKp30+ NK cells	↑↑↑	↔	↑
%NKp30+ CD56^bright^ cells	↑↑↑	↔	↑
%NKp30+ CD56^dim^ cells	↑↑↑	↔	↔
MFI NKp46 NK cells	↑↑↑	↔	↔
MFI NKp46 CD56^bright^	↑↑↑	↔	↔
MFI NKp46 CD56^dim^	↑↑↑	↔	↔
%CD57+ NK cells	↓↓↓	↔	↓↓↓
%CD57+ CD56^dim^ cells	↓↓↓	↔	↓↓
%NKG2A+ NK cells	↑↑↑	↔	↑↑↑
%NKG2A+ CD56^bright^ cells	↑↑↑	↔	↑
%NKG2A+ CD56^dim^ cells	↑	↔	↑↑
%CD94+ NK cells	↑↑↑	↔	↑↑
%CD94+ CD56^bright^ cells	↔	↔	↔
%CD94+ CD56^dim^ cells	↑↑↑	↔	↔
%CD107+ NK-medium	↔	↔	↔
%CD107+ NK cells-K562	↑↑	↔	↔
%CD107+ NK cells-Huh7.5	↔	↔	↔
%IFNg+ NK cells-medium	↔	↑	↔
%IFNg+ NK cells-K562	↑↑↑	↑	↔
%IFNg+ NK cells-Huh7.5	↔	↔	↔
%TNF+ NK cells- medium	↔	↑↑	↔
%TNF+ NK cells-K562	↑	↔	↔
%TNF+ NK cells-Huh7.5	↔	↑	↔

*↑/↓ - p<0.05 in the regression model.

↑↑/↓↓ - p<0.005 in the regression model.

↑↑↑/↓↓↓ - p<0.0001 in the regression model.

Further, also NKp46 expression was analysed. Expression intensity measured by mean fluorescence index (MFI) increased during PEG-IFNa therapy while ribavirin did not alter NKp46 levels (Table 2 and Fig. S2A).

TRAIL expression was analysed in all the three study arms. In accordance to previous data published by our group [20], changes in TRAIL expression correlated with interferon therapy, alone or in combination with ribavirin. Ribavirin monotherapy had no significant effect on TRAIL regulation (Fig. S2C).

This data confirms previous results [12], [14] that PEG-IFNa activates NK cells in vivo while we here demonstrate in addition that ribavirin does not cause any changes of NKp30 or NKp46 expression neither during monotherapy nor during combination therapy.

### Decline of Terminally Differentiated NK cells during PEG-IFNa Therapy

Various infections have been linked with alterations in frequencies of immature and differentiated NK cells [28]. Interferon alpha therapy may contribute to these changes. As we had observed alterations in NK cell subsets and NK cell activation during antiviral therapy of hepatitis C, we were next interested if percentages of NKG2A-positive and CD57-positive NK cells are influenced by treatment. Indeed, PEG-IFNa therapy was associated with a 1.5–2-fold increase of NKG2A-positive NK cells (35±4.7% day 42; 63±5.1% day 126 group A; 32±3.1% day 42; 53±4.2% day 126 group B; p<0.0001 in the regression model with no significant differences between both groups) (Fig. 2B and Table 2), paralleled by a decline of terminally differentiated CD57-positive NK cells (29±3.6% day 42; 21±4.1% day 126 group A; 37±4.8% day 42; 24±2.5% day 126 group B; p<0.0001) (Fig. 2C and Table 2). These changes were most obvious after 12 weeks of therapy but even the shorter course of 6 weeks of PEG-IFNa monotherapy (group C) led already to significant changes in expression of the two markers (Figure 2B and Table 2, p<0.0001 for both comparisons). Changes of NKG2A+/CD57− (immature) and NGK2A−/CD57+ (terminally differentiated) cells are shown in Figure S3 and Table 2. These effects could be related to PEG-IFNa exposure alone as ribavirin monotherapy had no apparent effects on NKG2A and CD57 expression.

We also examined the CD94 expression, known as a transmembrane protein which forms heterodimers on the NK cell surface either with NKG2A or NKG2C. Increase in the expression during interferon therapy and no significant effect during ribavirin monotherapy was noted (Fig. S2B).

Hence, a reshaping of the NK cell maturation status occurs during PEG-IFNa therapy of chronic hepatitis C with a loss of terminally differentiated NK cells and a relative increase of immature cells which is not influenced by ribavirin.

### Cytokine Production and Degranulation of NK Cells is Enhanced during PEG-IFNa Therapy

**Figure 3:**
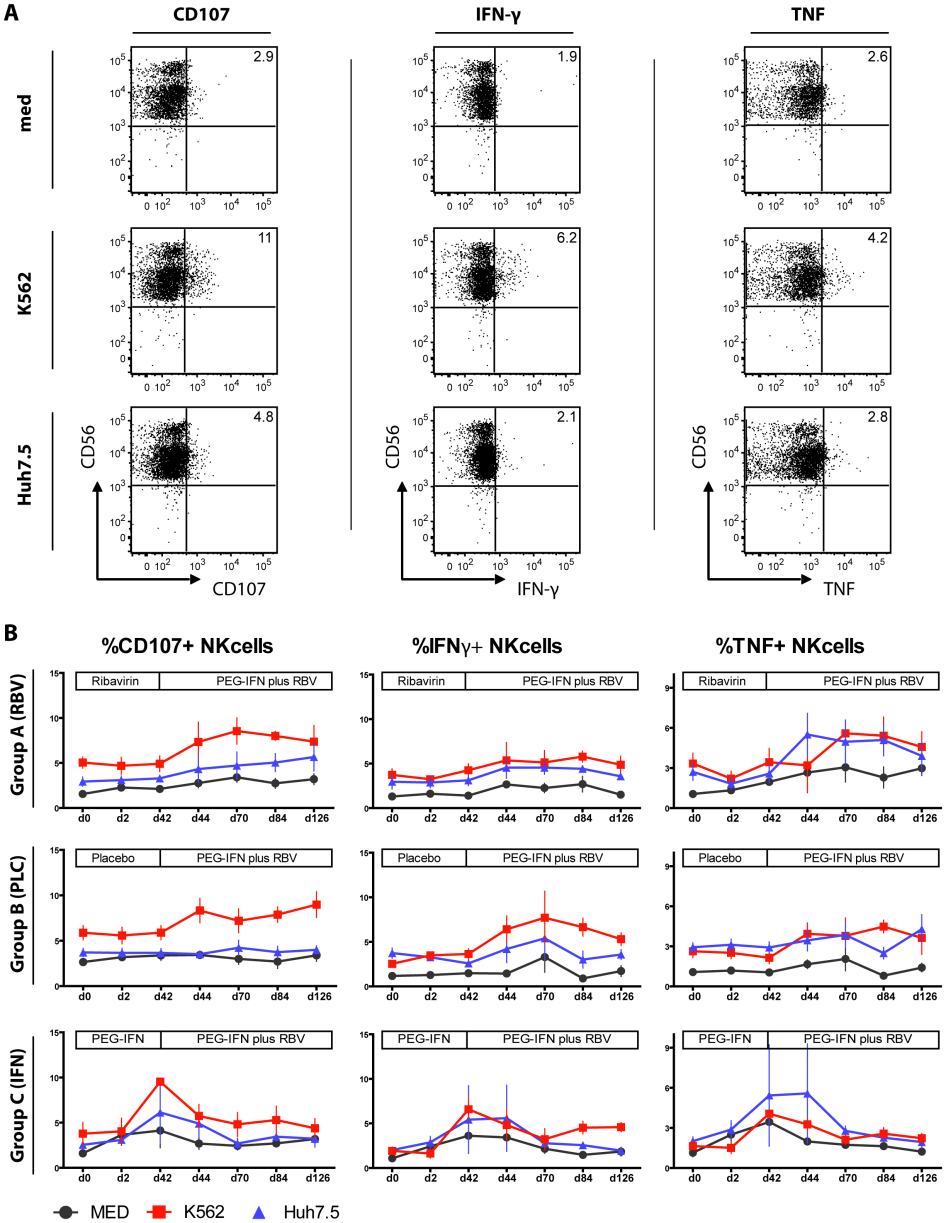
NK cell function during treatment. PBMC from HCV patients were cultured in medium alone or in co-culture with K562 or Huh7.5 target cells without any further cytokine stimulation. (A) Representative FACS plots for CD107a, IFNg and TNF expression on total NK cells without stimulation (MED) and after stimulation with target cells are shown. (B) Mean values of CD107a, IFNg and TNF expression on total NK cells without stimulation (MED- grey line) or upon stimulation with target cells - K562 (red line) or Huh7.5 (blue line) are shown.

As NK cells were activated by PEG-IFNa therapy, we next wondered if this would translate into altered effector function. Thus, we cultured PBMC obtained before and during therapy with different target cells including hepatoma cells. Of note and in contrast to several previous studies, PBMC were not further stimulated with additional cytokines in order to reflect the in vivo situation as close as possible. NK cell degranulation was determined by CD107a expression and cytokine production was tested by interferon-gamma (IFNg) and tumor necrosis factor (TNF) expression (Fig. 3A). As shown in Figure 3B, these effector functions were significantly enhanced by PEG-IFNa therapy within few days of therapy, in particular when assessed by co-culture with the NK-target cell line K562 (here p = 0.004 for CD107a; p<0.0001 for IFNg; p = 0.026 for TNF). Statistically, ribavirin monotherapy had also some effect on IFNg production and TNF expression until day 42 (Table 2) but this effect was only minor in magnitude (Fig. 3B). Interestingly, adding ribavirin after day 42 in patients which were first treated with PEG-IFNa alone (group C) did not enhance but rather shrinked degranulation and to some extent but not significantly also reduced cytokine production of NK cells (Fig. 3B).

Overall, these data clearly show that NK cell effector functions are enhanced by PEG-IFNa in vivo without any major additional effects of ribavirin.

### Ribavirin does not Alter NK Cell Effector Function in vitro

**Figure 4:**
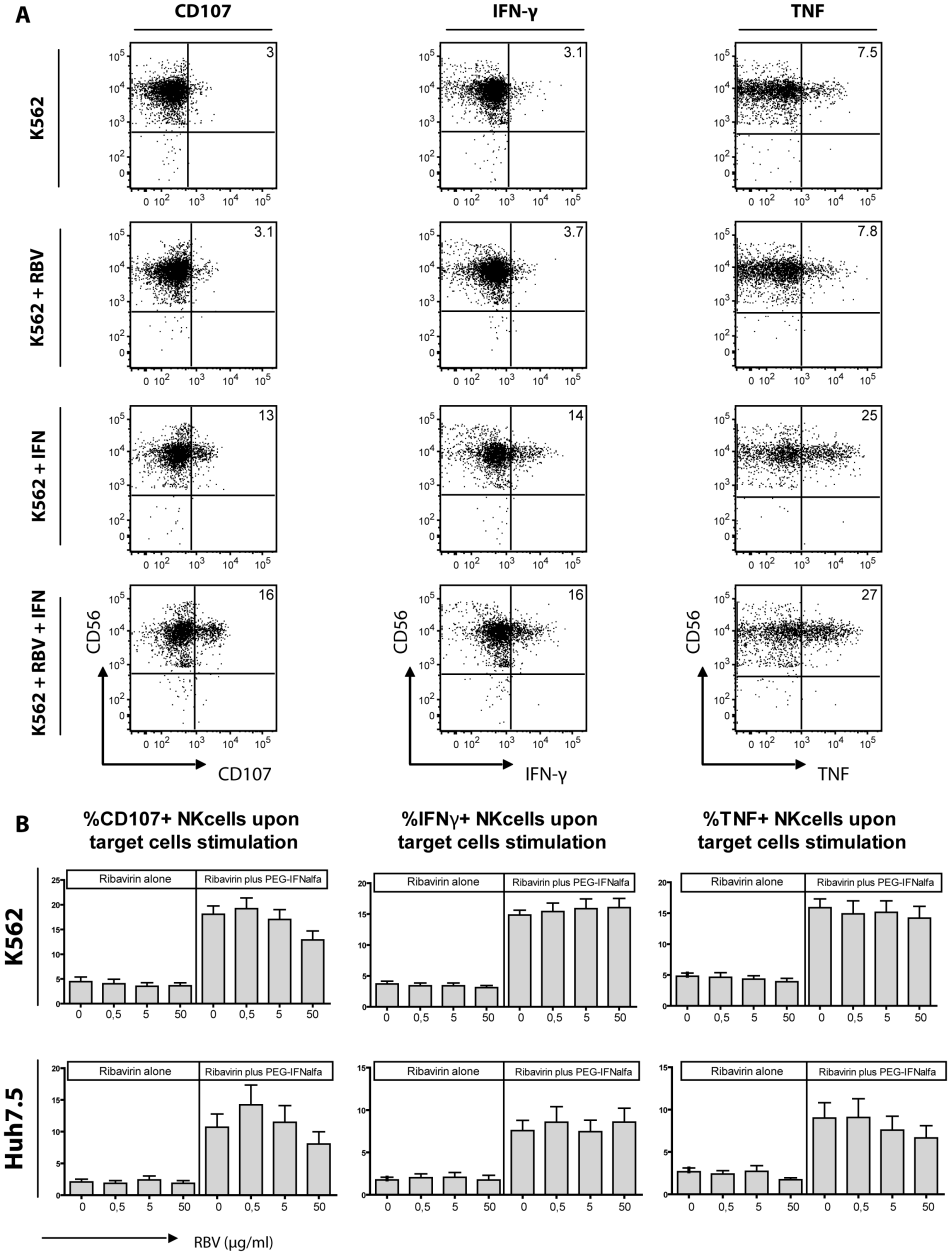
In vitro functionality of peripheral blood NK cells. NK cells from healthy individuals (n = 11) were stimulated for 6 hours with different concentration of RBV, PEG-IFNa and combination of both. (A) Representative FACS plots for NK cells stimulated with K562 target cells alone, with K562 target cells and RBV, with K562 target cells and PEG-IFNa, or with K562 target cells and combination of both are depicted. (B) Mean values of CD107a, IFNg and TNF expression on total NK cells upon stimulation with K562 target cells (B) and Huh7.5 hepatoma cells (C) from all healthy individuals.

Applying the above mentioned techniques, it is still possible that potential effects of ribavirin would have been overseen by ex vivo examination of cryopresevered PBMC. Thus, we aimed to investigate if ribavirin alters cytotoxicity or cytokine production of NK cells in vitro. Co-cultering NK cells with both K562 and Huh7.5 cells in presence of a wide range of ribavirin concentrations had no effect at all on the respective effector functions (Fig. 4). Similarly, ribavirin did not enhance CD107 expression, IFNg or TNF production induced by PEG-IFNa. In contrast, higher concentrations of ribavirin even reduced NK cell degranulation but not IFNg production. Adding IL-12/IL-15 to culture conditions yielded similar results as RBV exposure did not have any obvious effect on NK function even in the presence of IL-12 and IL-15 (Fig. S4). Thus, there was no evidence that potentially suboptimal assay conditions was hiding effects of ribavirin on NK cells.

Thus, these in vitro data support the observations from the clinical trial that ribavirin does not enhance NK cell effector function induced by PEG-IFNa but may even reduce NK cell degranulation.

### NK Cell Phenotype and Function are Associated with HCV RNA viral Load before and during Therapy

Having shown that PEG-IFNa treatment is associated with major changes in NK cell phenotype and function, we were interested to investigate whether the IFNa effect on NK cells translates in alterations of HCV viral loads and treatment response.

Overall, NK cell subsets distribution correlated with HCV RNA levels at baseline and during the 126 days of the study as higher frequencies of CD56^bright^ cells were associated with lower HCV RNA values (p = 0.0078). Similarly, negative associations were observed between HCV RNA levels and CD107 expression after co-culture with K562 (p = 0.03) and Huh7.5 cells (p = 0.04) as well as with IFNg expression (co-culture with K562, p = 0.0002).

Besides, we analysed the association between NK cell phenotype and function and the viral kinetic parameters which were fitted in by a mathematical model in [25]. Here, stronger responses to PEG-IFNa treatment which is characterized by the estimated efficacy parameter ε could be observed in patients with high baseline frequencies of CD57 positive cells in CD56^dim^ cells (p = 0.0451) and low frequencies of CD94 positive cells on CD56^ bright^ cells (p = 0.0044).

## Discussion

We here show that interferon alpha-based antiviral therapy of chronic hepatitis C is associated with major changes in the NK cell pool including an increase of CD56^bright^ cells, loss of terminally differentiated cells and an overall activation of the NK cell phenotype. Moreover, NK cell function both in terms of cytotoxicity and cytokine production was significantly enhanced during therapy. Importantly, we had the unique chance to distinguish potential effects of PEG-IFNa and ribavirin on NK cell phenotype and function in this trial. Of note, ribavirin did not contribute to the stimulatory effects of PEG-IFNa and may even suppress some effector functions of NK cells. Thus, the increased efficacy of combination therapy cannot be explained by additive or even synergistic effects of both drugs on NK cells.

It is well established that type I interferons activate NK cells [29]. Since hepatitis C virus infection is treated with high doses of interferon alpha, the in vivo effect of prolonged exposure to type I interferons can be studied prospectively in patients with chronic hepatitis C. Thus, findings in patients treated for HCV infection may also have implications for other chronic inflammatory conditions where type I interferon levels are elevated. Few studies published in recent years demonstrated already some evidence that treatment with pegylated interferon alpha and ribavirin induces a shift in the NK cell subset distribution with increases of CD56^bright^ cells within the first 12 weeks of therapy [12], [30]. We here provide a more detailed analysis of NK cell subset kinetics and show a constant decline of CD56^dim^ cells starting already about 2 weeks after initiating therapy. This effect could be attributed specifically to PEG-IFNa as 6 weeks of ribavirin monotherapy had no effect on the frequency of CD56^bright^ or CD56^dim^ cells. Of note, altered frequencies of CD56^bright^ cells are a hallmark of various inflammatory conditions in humans including patients with HIV infection [31].

As expected, PEG-IFNa treatment induced an activatory phenotype of NK cells. NKp30 and NKp46 levels increased during therapy which is in line with previous reports showing marked changes in NK cell phenotype as early as 6–48 hours after the first dose of PEG-IFNa [12]. We here demonstrate that both markers gradually increased with continued PEG-IFNa therapy for up to 18 weeks. This activation was also associated with a loss of terminally differentiated NK cells. The relative increase in immature cells is likely to be caused by apoptosis of differentiated cells since NK cell maturation is considered irreversible [32] on the other hand migration of these cells to other organs including the liver could be an alternative explanation. On the other hand, immature cells may preferentially proliferate in response to PEG-IFNa exposure as it has been shown for other cytokines including IL-2, IL-12 or IL-15 [33]–[35]. Even though ribavirin has been suggested to shift cytokine responses towards a Th1-type profile [22], ribavirin did not alter IFNa-induced reshaping of the NK cell pool and had also no effects on its own during monotherapy. Thus, this mechanism of potential immunomodulation by ribavirin does not seem to be of major importance, at least concerning NK cell maturation and proliferation.

We recently showed that in vitro stimulation of NK cells with interferon alfa increased degranulation and cytokine production after co-culture of NK cells with K562 cells and hepatoma cells [27]. Following this in vitro study, we here addressed the question if NK cells studied directly ex vivo after treatment with PEG-IFNa without any further in vitro exposure to interferon alfa would also show enhanced effector function. Previous studies investigating NK cells during antiviral therapy further stimulated the cells in vitro with different cytokines [12], [36]. Thus, we here demonstrate for the first time that unmanipulated NK cells exposed only in vivo to interferon alpha display indeed stronger effector functions which was detectable as early as 2 days after first PEG-IFNa injection. Importantly, CD107a expression as a marker of degranulation and also IFNg production of NK cells were inversely correlated with HCV RNA levels during therapy suggesting a direct role of activated NK cells in the control of HCV replication during PEG-IFNa therapy.

Potential mechanisms how interferon alpha and ribavirin may influence effector functions of NK cells may involve regulation of STAT (Signal Transducers and Activators of Transcription) molecule expression and phosphorylation. Interferon alpha has been shown to alter the ratio of different STATs in NK cells with increases in STAT1 levels vs. reduction of STAT4 expression [37]. STAT1 expression and induction of STAT1-phosphorylation has been shown to be enhanced in NK cells of patients with hepatitis C which is then further increased by IFNa therapy, potentially leading to a more cytotoxic phenotype [38]. Other researchers suggested that ribavirin may further enhance IFNa-induced STAT1 and STAT3 phosphorylation in hepatocytes [39] but considering the lack of synergistic effects in this study it is unlikely that ribavirin and IFNa act in this way on NK cells. In contrast, ribavirin may even reduce phosphorylation of various signalling molecules including STAT1 in human hepatoma cells [40]. Similar mechanisms could explain the minor counterregulatory effects of ribavirin which was evident in direct ex vivo determination of NK cell degranulation of treated patients but also in the in vitro studies stimulating PBMC from healthy donors with ribavirin and PEG-IFNa.

Even though it is well established that ribavirin increases virological response to IFNa-based therapy of chronic hepatitis, the detailed modes of action are still a matter of debate [21], [41]. Collectively, the data presented in this study clearly argue against a major immunomodulatory role or ribavirin involving NK cells which is based on both in vitro observations but also on ex vivo examination of treated patients. Ribavirin monotherapy was associated with declines in HCV RNA and alanin aminotransferase (ALT) levels [25] but these responses did not correlate with NK cell markers and NK cells function (data not shown). However, numbers were small and we therefore cannot exclude completely potential association. The lack of ribavirin effects on NK cells in vitro was also unlikely to be due to suboptimal cell culture conditions as even adding IL-12 and IL-15 did not reveal any different results. Overall, these findings are of potential importance for future interferon-free therapies of hepatitis C still containing ribavirin. Immune control is likely to play an important role to prevent relapse after treatment with distinct all-oral antiviral therapies. E.g., more than 30% of patients treated with sofosbuvir and ribavirin without interferon alpha experienced an HCV RNA relapse in recently published phase III trials [42]. Very preliminary data from a pilot study performed at the NIH suggested that impaired T cells but also NK cell function may be restored in part during sofosbuvir therapy which was associated with a lower likelihood of HCV relapse [43]. Based on the data from our study, we do not think that ribavirin did directly contribute to the changes in immune cell function observed in the NIH trial but this certainly warrants further investigation in future studies.

In conclusion, antiviral activities of RBV cannot be linked to apparent phenotypical or functional changes of NK cells. PEG-IFNa activates NK cells possibly contributing to virological responses independently of RBV. The role of NK cells during future IFN-free combination therapies including RBV remains to be determined.

## Supporting Information

Figure S1 **Phenotypical changes of NK cells during treatment with RBV, PEG-IFNa and combination therapy.** PBMC were stained directly ex vivo. Single patient courses for expression of (A) NKp30 (B) NKG2A and (C) CD57 on CD56^bright^ and CD56^dim^ cells are shown. Percentage of positive cells as well as fold change as compared to baseline expression are represented.
(TIF)

Figure S2 **Phenotypical changes during treatment with RBV, PEG-IFNa and combination therapy.** PBMC were stained directly ex vivo. Single patient courses for expression of (A) NKp46 (B) CD94 and (C) TRAIL on NK cells are shown. MFI or percentage of positive cells as well as fold change as compared to baseline expression are represented.
(TIF)

Figure S3 **Phenotypical changes over time during treatment with RBV, PEG-IFNa and combination therapy.** PBMC were stained with the respective markers directly ex vivo. Mean values for the frequencies of (A) immature NKG2A+CD57- and (B) mature NKG2A-CD57+ NK cells are shown.
(TIF)

Figure S4 **In vitro functionality of peripheral blood NK cells.** NK cells from healthy individuals (n = 10) were stimulated for 6 hours with different concentrations of RBV alone or in combination with IL-12/IL-15 and interferon alpha as indicated. Cells were co-cultured with K562 target cells. IFNg production on total NK cells was analysed as indicated in materials and methods. There were no statistical differences between cells stimulated with IL-12/IL-15 and IL-12/IL-15 plus ribavirin.
(TIF)

Table S1 **Individual patient characteristics.**
(DOCX)

Methods S1 (DOCX)
