# Supplementary figures and images for: PEG-IFN Alpha but Not Ribavirin Alters NK Cell Phenotype and Function in Patients with Chronic Hepatitis C
Source: PLoS One. 2014 Apr 21;9(4):e94512. doi: 10.1371/journal.pone.0094512 (PMC3994015; doi:10.1371/journal.pone.0094512)

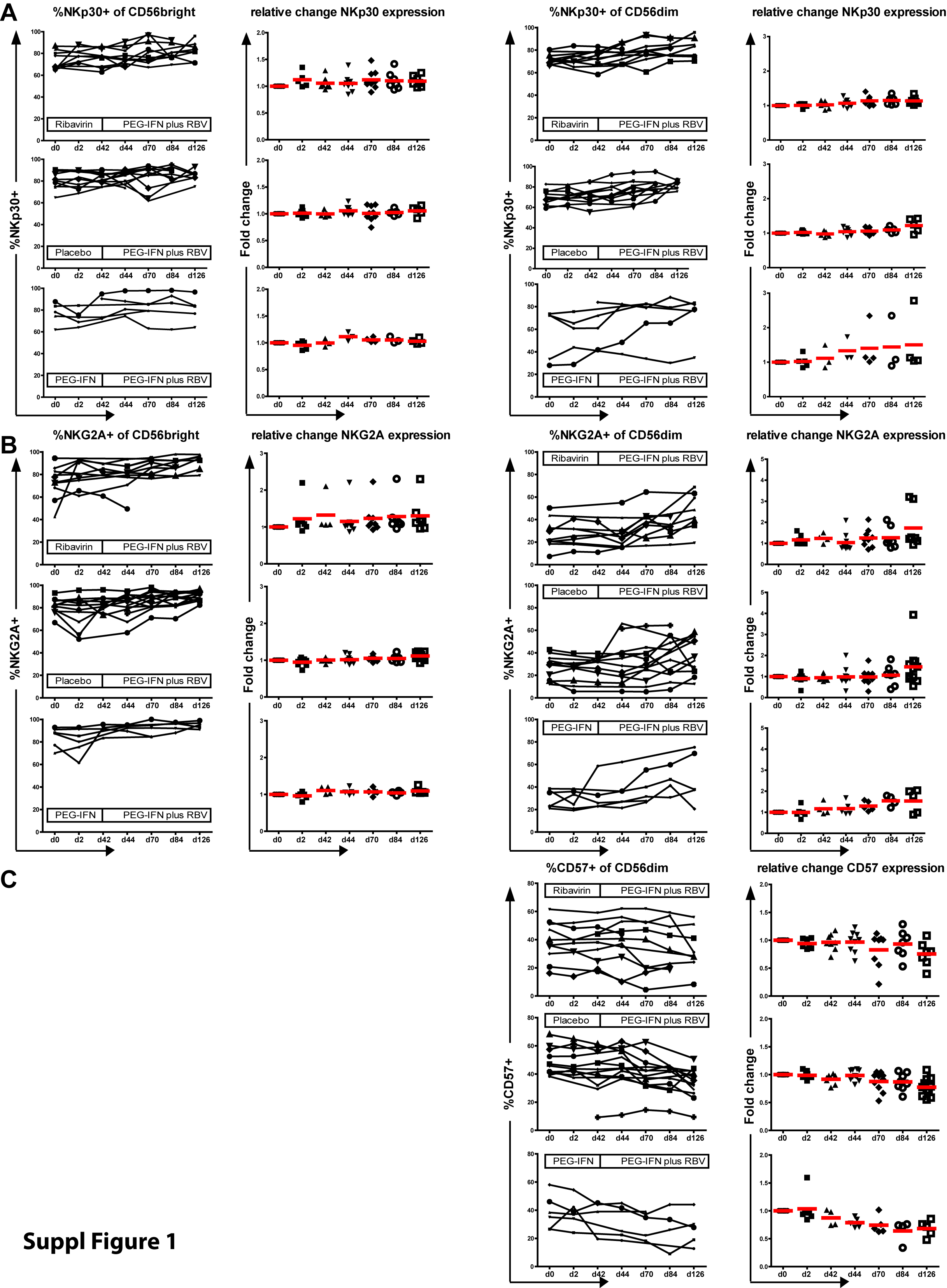

Supplement: Figure S1 — Phenotypical changes of NK cells during treatment with RBV, PEG-IFNa and combination therapy. PBMC were stained directly ex vivo. Single patient courses for expression of (A) NKp30 (B) NKG2A and (C) CD57 on CD56bright and CD56dim cells are shown. Percentage of positive cells as well as fold change as compared to baseline expression are represented. (TIF) [file pone.0094512.s001.tif]

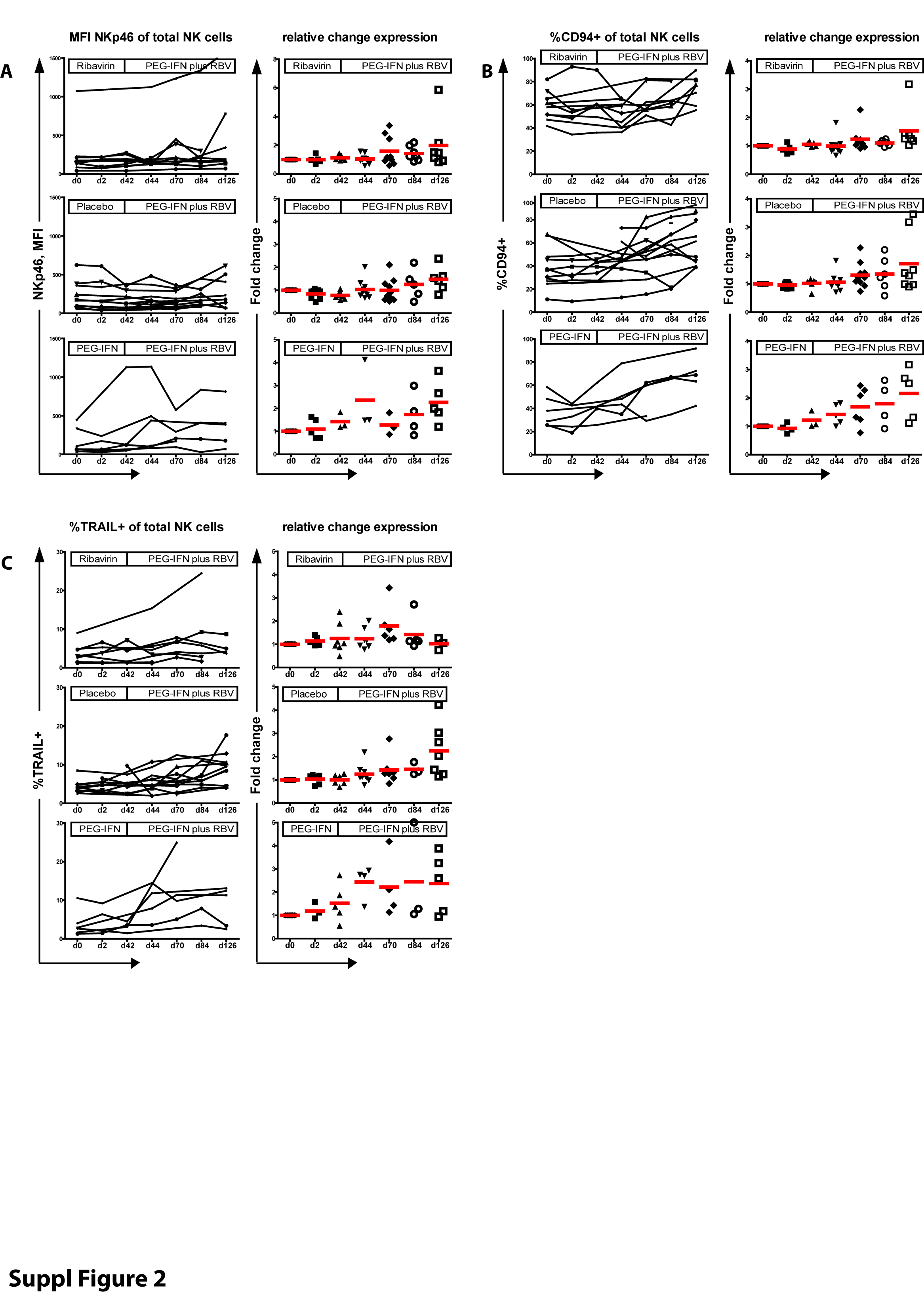

Supplement: Figure S2 — Phenotypical changes during treatment with RBV, PEG-IFNa and combination therapy. PBMC were stained directly ex vivo. Single patient courses for expression of (A) NKp46 (B) CD94 and (C) TRAIL on NK cells are shown. MFI or percentage of positive cells as well as fold change as compared to baseline expression are represented. (TIF) [file pone.0094512.s002.tif]

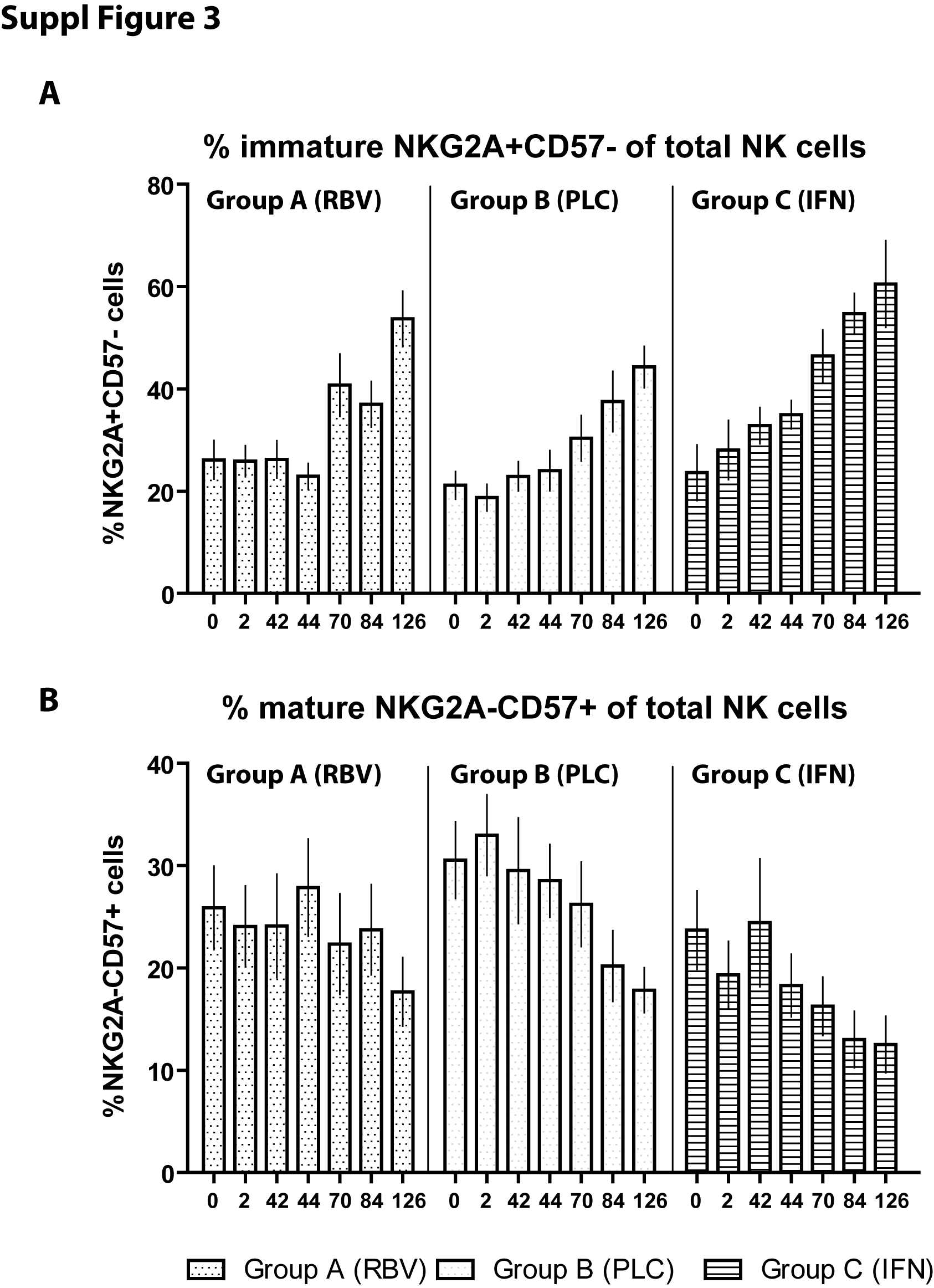

Supplement: Figure S3 — Phenotypical changes over time during treatment with RBV, PEG-IFNa and combination therapy. PBMC were stained with the respective markers directly ex vivo. Mean values for the frequencies of (A) immature NKG2A+CD57- and (B) mature NKG2A-CD57+ NK cells are shown. (TIF) [file pone.0094512.s003.tif]

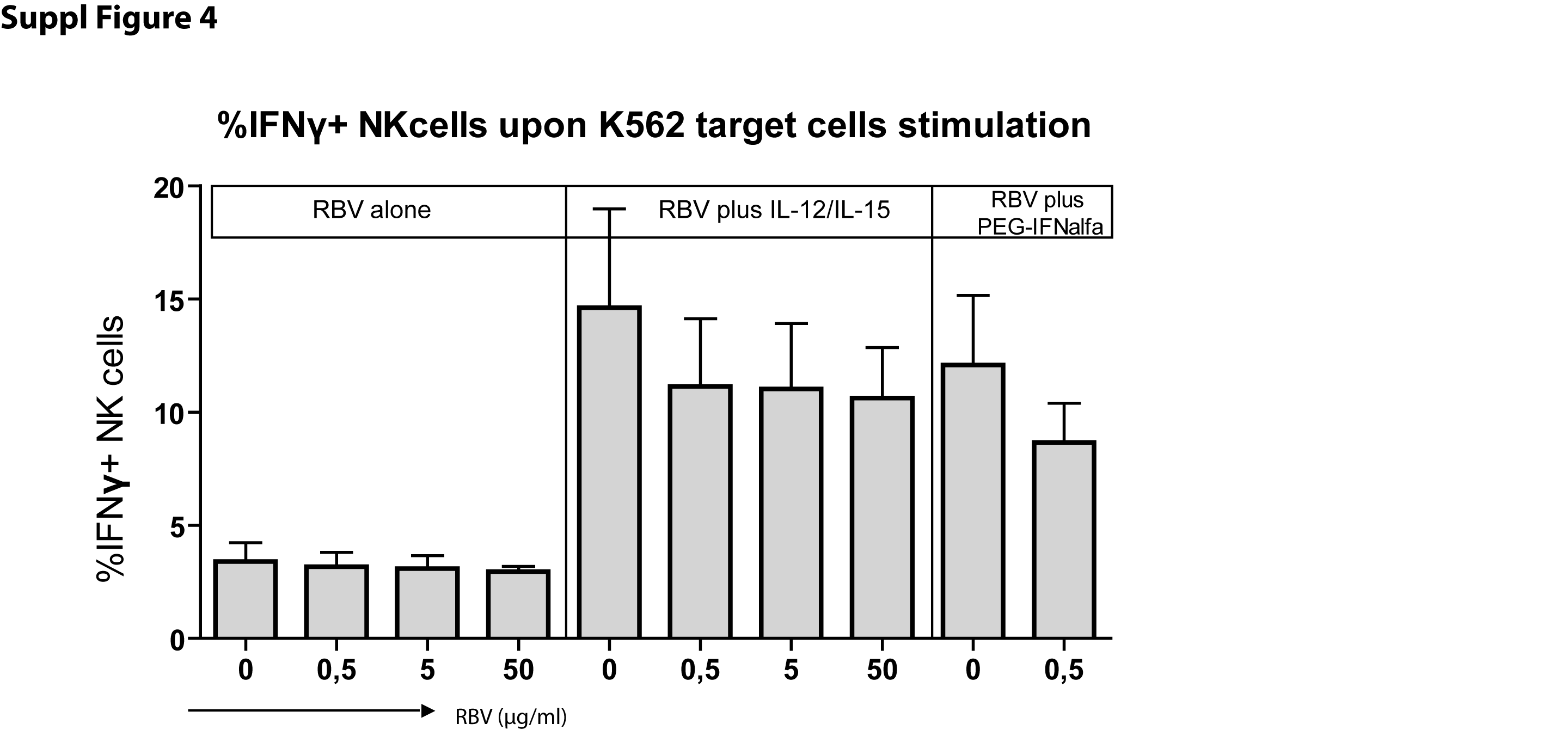

Supplement: Figure S4 — In vitro functionality of peripheral blood NK cells. NK cells from healthy individuals (n = 10) were stimulated for 6 hours with different concentrations of RBV alone or in combination with IL-12/IL-15 and interferon alpha as indicated. Cells were co-cultured with K562 target cells. IFNg production on total NK cells was analysed as indicated in materials and methods. There were no statistical differences between cells stimulated with IL-12/IL-15 and IL-12/IL-15 plus ribavirin. (TIF) [file pone.0094512.s004.tif]
