# Supplementary material for: PEG-IFN Alpha but Not Ribavirin Alters NK Cell Phenotype and Function in Patients with Chronic Hepatitis C
Source: PLoS One. 2014 Apr 21;9(4):e94512. doi: 10.1371/journal.pone.0094512 (PMC3994015; doi:10.1371/journal.pone.0094512)
Supplement: Table S1 — Individual patient characteristics. (DOCX) [file pone.0094512.s005.docx]

**Table S1**. Individual patient characteristics.

| **Patient N** | **Therapy Group** | **Response**  **TW4 PEG-IFNa-RBV** | **Response**  **TW12 PEG-IFNa-RBV** | **Outcome** | **HCV RNA d0, IU/ml** | **ALT d0, U/ml** | **AST d0, U/ml** | **Ag, y** | **Sex** | **BMI** |
| --- | --- | --- | --- | --- | --- | --- | --- | --- | --- | --- |
| A-P1 | A (RBV) | neg | neg | SVR* | 1.0x10^5^ | 87 | 33 | 27 | M | 25.9 |
| A-P2 | A (RBV) | neg | neg | SVR | 6.1x10^6^ | 74 | 48 | 49 | F | 22.3 |
| A-P3 | A (RBV) | pos | neg | NR* | 1.7x10^6^ | 65 | 92 | 66 |  | 26.5 |
| A-P4 | A (RBV) | pos | pos | NR | 4.4x10^6^ | 187 | 233 | 46 | M | 33.2 |
| A-P5 | A (RBV) | neg | neg | Lost to FU | 6.0x10^5^ | 98 | 48 | 49 | M | 30.9 |
| A-P6 | A (RBV) | nd | nd | discontinuation | 1.5x10^6^ | 55 | 52 | 54 | F | 21.5 |
| A-P7 | A (RBV) | pos | neg | SVR | 1.0x10^6^ | 48 | 32 | 42 | F | 28.3 |
| A-P8 | A (RBV) | pos | neg | SVR | 3.0x10^5^ | 45 | 31 | 40 | F | 20 |
| A-P9 | A (RBV) | pos | pos | NR | 1.5x10^6^ | 31 | 48 | 47 | M | 21 |
| A-P10 | A (RBV) | pos | neg | REL* | 5.4x10^6^ | 48 | 44 | 56 | F | 33.6 |
| A-P11 | A (RBV) | pos | neg | NR | 5.0x10^5^ | 32 | 26 | 46 | F | 32.8 |
| B-P1 | B (PLC) | pos | pos | NR | 1.4x10^7^ | 64 | 53 | 51 | F | 25.4 |
| B-P2 | B (PLC) | pos | neg | SVR | 2.0x10^5^ | 62 | 35 | 39 | F | 20.3 |
| B-P3 | B (PLC) | pos | pos | NR | 6.9x10^7^ | 107 | 75 | 51 | F | 24.3 |
| B-P4 | B (PLC) | pos | pos | NR | 2.2x10^6^ | 42 | 42 | 70 | F | 24.4 |
| B-P5 | B (PLC) | pos | pos | NR | 7.0x10^5^ | 128 | 95 | 64 | F | 24.7 |
| B-P6 | B (PLC) | neg | neg | SVR | 1.0x10^5^ | 40 | 34 | 68 | F | 35.6 |
| B-P7 | B (PLC) | neg | neg | SVR | 3.2x10^6^ | 63 | 35 | 56 | F | 32 |
| B-P8 | B (PLC) | pos | pos | SVR | 4.7x10^6^ | 114 | 119 | 69 | M | 26.6 |
| B-P9 | B (PLC) | neg | neg | SVR | 1.0x10^5^ | 42 | 31 | 36 | F | 23.5 |
| B-P10 | B (PLC) | pos | pos | REL | 3.0x10^5^ | 135 | 73 | 36 | M | 30.3 |
| B-P11 | B (PLC) | pos | pos | REL | 7.7x10^6^ | 172 | 150 | 57 | M | 25.1 |
| B-P12 | B (PLC) | pos | pos | REL | 1.8x10^7^ | 94 | 57 | 46 | M | 28.5 |
| B-P13 | B (PLC) | neg | neg | SVR | 2.0x10^5^ | 40 | 28 | 22 | F | 23.7 |
| C-P1 | C (IFN) | neg | neg | NR | 6.9x10^6^ | 53 | 38 | 23 | M | 23.5 |
| C-P2 | C (IFN) | pos | pos | NR | 9.2x10^6^ | 159 | 109 | 55 | M | 23.4 |
| C-P3 | C (IFN) | pos | pos | NR | 2.8x10^6^ | 66 | 56 | 51 | F | 21.4 |
| C-P4 | C (IFN) | pos | pos | REL | 4.1x10^6^ | 59 | 67 | 70 | M | 29.4 |
| C-P5 | C (IFN) | pos | neg | SVR | 6.9x10^6^ | 116 | 87 | 55 | M | 29 |
| C-P6 | C (IFN) | neg | neg | SVR | 2.3x10^6^ | 97 | 75 | 47 | F | 23.5 |

*

SVR = sustained viral response

NR = nonresponse

REL = relapse

FU = follow-up

TW = therapy week

PLC = placebo

nd = not determined
