# Supplementary material for: PEG-IFN Alpha but Not Ribavirin Alters NK Cell Phenotype and Function in Patients with Chronic Hepatitis C
Source: PLoS One. 2014 Apr 21;9(4):e94512. doi: 10.1371/journal.pone.0094512 (PMC3994015; doi:10.1371/journal.pone.0094512)
Supplement: Methods S1 — (DOCX) [file pone.0094512.s006.docx]

**Supplementary Information Methods**

PBMC were stained with ethidium monoazide, human anti-CD3-APC-eFluor 780 (eBioscience) to exclude T cells, and human anti-CD56-PeCy7 and human anti-CD16-PeCy5 (BD Biosciences) to identify NK cells. Additionally, cells were stained with saturating amounts of the desired mouse-derived anti-human monoclonal antibodies for flow cytometry: anti-CD57-FITC (Beckman Coulter), anti-NKp30-Alexa Fluor 647 (BD Biosciences), anti-NKp46-PE (BD Biosciences), anti-NKG2A-APC (Beckman Coulter), human anti-CD94-APC (BD Biosciences).

NK cells cytotoxicity and cytokine production was assessed by upregulation of cell surface CD107a expression after co-culture with different target cells. Cryopreserved PBMC were thawed and cultured at 2,5x10^6^ cells/ml at 37°C in complete RPMI medium (RPMI-1640, 10% human AB serum (Cambrex, East Rutherford, NJ, USA), 100U/ml Penicillin, 0.1mg/ml Streptomycin, 1% MEM non-essential amino acids, 1% sodium pyruvate and 2mM L-Glutamine (PAA Laboratories GmbH, Parching, Austria)) without any exogenous added cytokines. Cells were shortly rested and then stimulated in the presence of human anti-CD107a-PE antibody with either (1) major histocompatibility complex (MHC) class I-negative K562 target cells or (2) hepatoma Huh7.5 cells (Life Technologies, Grand Island, NY, USA) in an effector to target ratio 10:1 or (3) in complete medium without any target cells. After one hour Brefeldin A (BD Biosciences) was added for another 5 hours at 37°C. Finally PBMC were washed, surface staining for degranulation was performed. For cytokine production cells were fixed and permeabilised using the Cytoperm/Wash Buffer Kit (BD Biosciences) and stained intracellularly with human anti-IFNg-FITC (BD Biosciences) and human anti-TNF-APC (BD Biosciences). After further washing cells were measured by flow cytometry (FACS Canto II using FacsDiva version 4.1 ((BD Biosciences)) and analyzed using FlowJo Version 6.7 (Tree Star Inc., Ashland, OR) software.
